# Comparing hospital stay and patient satisfaction in a resource poor setting using conventional and locally adapted negative pressure wound dressing methods in management of leg ulcers with split skin grafts: a comparative prospective study

**DOI:** 10.11604/pamj.2020.36.105.19961

**Published:** 2020-06-18

**Authors:** Charles Chidiebele Maduba, Ugochukwu Uzodimma Nnadozie, Victor Ifeanyichukwu Modekwe, Ezekiel Uchechukwu Nwankwo

**Affiliations:** 1 Division of Plastic Surgery, Alex Ekwueme Federal University Teaching Hospital, Abakaliki, Ebonyi State, Nigeria,; 2 Nnamdi Azikiwe University Teaching Hospital, Nnewi, Anambra State, Nigeria

**Keywords:** Conventional gauze dressing, length of stay, negative pressure dressing, patient satisfaction

## Abstract

**Introduction:**

chronic leg ulcers cause a prolonged hospital stay with devastating effects on the patients. Several modifiable factors are taken care of to reduce the duration of stay. A further measure to hasten wound bed preparation pre-grafting and to hasten graft healing post-grafting is with negative pressure dressing.

**Methods:**

sixty-two patients were placed in two groups of 31 cases each. The wound beds were prepared with negative pressure apparatus locally adapted with suction machine for group A and with conventional gauze dressing using 5% povidone iodine soaks for group B. Grafted wound was also dressed similarly for the respective groups. Grafts were inspected on the 5^th^ post-operative day and were determined with planimeter grid. Grafts were monitored until completely healed and patients were discharged. Satisfaction and length of stay were determined at discharge.

**Results:**

the mean hospital stay pre-grafting and post-grafting were 12.2 (±8.64) days and 13.6 (±2.03) days respectively for the negative pressure dressing and 28.8 (±30.9) days and 21.8 (±21.97) days respectively for the traditional dressing group. These differences with p values of 0.038 for the pre-grafting stay and 0.006 for the post-grafting stay were statistically significant. The patients managed with negative pressure dressing also recorded greater satisfaction with the process and the outcome.

**Conclusion:**

negative pressure dressing contributes significantly to reducing the length of hospital stay in chronic leg ulcers both in wound bed preparation and in graft healing resulting to better patient satisfaction than in patients treated with conventional gauze dressing and 5% povidone iodine soaks.

## Introduction

Chronic leg ulcers are defined traditionally as wounds that fail to heal after 4 weeks of optimal management or fail to show 20-40% reduction in size after 2-4 weeks of optimal therapy [1]. They constitute a major burden to the patient and account for long hospital stay [2]. They occur worldwide for various reasons which vary from one socio-economic status to another. It is mainly due to chronic venous insufficiency in developed countries but due to poorly managed traumatic injuries in developing countries [3]. Chronic leg ulcers affect all age groups but show a bimodal peak pattern affecting ages 30-39 years and 50-69 years in a single centre report in Nigeria [4]. This has a very untoward effect on economic productivity of the patients especially the first peak. In addition ulcers being a microcosm of the victim have a far reaching effect on the patient emotionally, nutritionally, socially and economically [5]. The additional burden of hospital stay and consequent cost of care take a big toll on the patient. It is not only a problem to the patient; the hospital too may be stretched by the burden of in-patient care [6]. It is known that chronic leg ulcers are on increase because of improved quality of life and longevity with their attendant chronic venous insufficiency and atherosclerosis causing venous and arterial ulcers respectively [5].

Several factors could be responsible for the duration of hospital stay. These are factors that commonly affect the physiological reserve of the patients. Co-morbidities, malnutrition, anemia among others are usually considered to play a role in such patients [7]. The patients enrolled in the study therefore all had normal serum albumin, adequate hematocrit and normal sugar levels. The need to further reduce the length of hospital study after the modifiable factors have been handled necessitates this study. There are several options of wound cover of which split-thickness skin graft is one. It is indeed the most common surgical procedure and the gold standard in safe and effective management chronic leg wounds [8]. Prior to skin grafting the wound bed is prepared to rid it of infection and achieve a bed that could optimally support skin graft take. This is usually done with conventional gauze dressing with 5% iodine in our centre. Other solutions and salves also used depending on clinical judgment. This form of dressing is also used for dressing of the skin graft intra-operatively and post-operatively. Graft take has been variable using the conventional gauze dressing. Poor graft take invariable contributes to the hospital stay. Together with donor site morbidity poor graft healing contributes to prolonged hospital stay. Donor site factors are considered in the patient who continued and completed the study. The aim of this study is to compare how the negative pressure dressing affects patient hospital stay both pre-grafting and post-grafting and to evaluate how the patients were satisfied in each form of dressing.

## Methods

This was a one year prospective comparative study of patients with leg and foot ulcers managed with split-thickness skin grafting at National Orthopedic Hospital Enugu, Nigeria. It is a major referral centre for plastic surgery in Nigeria. It serves patients from Southeast, South-south states and few states of the North-central region of Nigeria. Ethical approval was obtained from the hospital´s research and ethics committee. Sixty-two patients who met the inclusion criteria set for the study were enrolled. All sickle cell ulcers, poorly controlled diabetes mellitus with ulcers, malignant ulcers, arterial ulcers, ulcers with underlying chronic osteomyelitis and children below twelve years and patients who refused to be part of the study were excluded. Patients were grouped into two using convenient sampling. Each group had 31 patients who were treated respectively with conventional gauze dressing and negative pressure dressing. Basic investigations were done to rule out anemia, malnutrition and electrolyte, urea, creatinine and sugar imbalances. All wounds were debrided and made clean before patient is considered to begin participation in the study. Patients in the group A were treated with locally adapted negative pressure apparatus which was made of polyurethrane foam with size 18 feeding tube with multiple perforations, insinuated through the foam. There is no conflict of interest and the research extra costs were borne by the investigators.

This is placed over the wound dressed with framycetin impregnated tulle gauze and a layer of povidone iodine soaked gauze. The dressing is further covered with airtight op-site and the feeding tube connected to a suction machine. The suction pressure of 100 mmHg was maintained for a period of 12 hours per day intermittently running as one hour on and one hour off. Dressing was changed every 72 hours till the wound bed was optimal. Wound swab was sent for culture and sensitivity and target antibiotic therapy given as indicated. The group B patients were managed with conventional gauze dressing which comprised the tulle gauze contact layer, the povidone iodine gauze layer and the cotton wool/absorptive layer bandaged with crepe bandage. The dressings were done on alternate day basis till 4 days to surgery when daily dressing is commenced. Antibiotics were given as necessary. Other adjunct treatments such as use of vitamins and zinc supplements were same for all patients. Use of plaster of Paris back-slab was for selected patients with ulcers around the joints. Limbs were uniformly elevated in both groups. Regional or general anesthesia was used as deemed best for the patient by the anesthetists. Under anesthesia intravenous antibiotics were given and procedure carried out under strict asepsis. Skin was harvested with Watson knife set at the third mark setting aiming for an intermediate thickness graft.

Donor site hemostasis was achieved with 1:100,000 epineprine. The wound was dressed in three layers with tulle gauze, povidone iodine soaked gauze and cotton wool and covered with crepe bandages. The wound beds were then prepared with blunt scraping to remove the biofilms and washing with dilute hydrogen peroxide. The size of the wound was determined by tracing with sterile paper and transferring to planimeter grid to determine the size in cm^2^. The grafts were then fenestrated, inset on the wound and anchored with skin staples. The chosen dressing method was applied on the graft. The same negative pressure of 100 mmHg used on the wound pre-grafting was maintained post-grafting. Graft inspection was done on the 5^th^ post-operative day. Areas of graft loss were traced with sterile paper and transferred to the planimeter grid. Its percentage of the original wound size was calculated to determine the percentage graft take. Graft infection was confirmed by culture where clinically suspected and treated based on sensitivity. Patients were discharged when the grafts had healed. Duration of stay was determined at discharge. Patient satisfaction was also assessed on discharge by an independent blinded research assistant. Data obtained were analyzed IBM SPSS windows (version 21.0; IBM Corp., Armonk, New York). P-values less than 0.05 were considered statistically significant.

## Results

There were 62 patients in all with males comprising 62.9% and females comprising 37.1%. The male to female ratio was 1.7:1. The mean hematocrit was 34.7 (±2.62)% in the negative pressure dressing group and 35.1 (± 3.80)% in the conventional dressing group. Serum albumin level had average values of 3.67 (±0.588) mg/dl for group A and 3.42 (±0.571)mg/dl for group B. P-value was 0.670 for hematocrit and 0.093 for the albumin. In Table 1, the duration of stay pre-grafting was 12.2 (±8.64) days for the negative pressure group and 28.77 (±30.9) days for the conventional wound dressing method group. P-value was 0.006 which was statistically significant. The length of hospital stay post grafting is presented in Table 2. It shows respective mean values of 13.6 (±2.03) for group A and 21.8 (±21.97) for the group B. The p-value of 0.038 was statistically significant. Table 3 compares patient satisfaction in both forms of dressing. The negative pressure dressing group had significantly better satisfaction than the patients who had conventional wound dressing.

**Table 1:** independent samples test on the mean hospital pre-graft stay duration of both forms of dressing

	Mean duration of pre-graft hospital stay	
Negative pressure dressing	12.2(±8.64)	p-value
Traditional wound dressing	28.77(±30.9)	.006

**Table 2:** comparing duration of hospital stay post-graft in both forms of dressings

Type of dressing	Mean duration in days	p-value
Negative pressure dressing	13.6(±2.03)	0.038
Traditional wound dressing	21.8(±21.97)	

**Table 3:** comparing patients’ satisfaction in both forms of dressings

Type of dressing	Satisfaction	Frequency	Chi-square	p-value
Negative pressure dressing	Very satisfied	31		
	Moderately satisfied	0		
	Fairly satisfied	0		
	Not satisfied	0		
Traditional wound dressing	Very satisfied	3		
	Moderately satisfied	22		
	Fairly satisfied	6	20.194	.000
	Not satisfied	0		

## Discussion

Prolonged hospital stay in chronic leg ulcers has been found to be due to a lot of modifiable factors which include anemia, malnutrition and electrolyte and glucose imbalance among others. These factors were optimal in the patients used for the study or otherwise corrected. This was done to eliminate the confounding factors that affect the length the of hospital stay in the patients. The study shows a significant difference in the hospital stay both pre-grafting and post-grafting between the two groups. The mean stay of patients in the negative pressure dressing group pre-grafting was 12.2 (±8.64) days as compared to 28.77 (±30.9) days in the traditional wound dressing group. The p-value was 0.006 which was statistically significant. This mean hospital stay of 12.2 days in the negative pressure group is similar to the findings by Jiburum *et al*. which showed a pre-grafting period of about 14 days in wound managed with vacuum-assisted wound closure. Compared to a mean period of about 28.8 days in the traditional wound dressing, which is about 2.4 times longer, the impact on the patient socially and economically would be enormous. The reasons adduced for faster wound bed preparation and consequent reduced hospital stay in the pre-grafting period were: the ability of the negative pressure dressing to enhance faster wound bed preparation through increase in flow blood by about 4 times the basal level, reduction in local oedema, decrease in bio-burden and increased granulation tissue formation by about 40-103% the basal level [9].

The post-grafting mean hospital duration of stay was 13.6 (± 2.03) days for the negative pressure dressing while for the traditional dressing it was 21.8 (±21.97) days. The p-value was 0.038 which is statistically significant. So, in the post-grafting period also, there is a significant decrease in the hospital stay. The traditional wound dressing with post-grafting stay of about 21.8 days compared to negative pressure dressing with duration of about 13.6 days implies that the traditional wound dressing group stayed as much as 1.6 times the negative pressure group. This would also have a huge impact on the patient socio-economically. The shorter post-grafting stay is as a result of better graft take and minimizing of complications. This also agrees with position of Moshim M *et al*. which showed maximum hospital stay of 22 days in the traditional wound dressing and the 9 days in the negative pressure dressing group with a statistically significant p-value. They also discovered the impact of this on the cost of care which they found to be 22 times more in the traditional wound dressing group while using a locally adapted negative wound dressing method [10]. It was also discovered by Petkar *et al*. that in addition to improved graft take of about 10% difference between the negative pressure dressing group and the conventional dressing, there was reduction in the duration of graft dressing requirements.

This also reduced post grafting stay in the hospital [11]. Saaiq *et al*. attributed the reduced hospital stay to better graft take and faster wound healing time. In their study over 90% of the negative pressure dressing had their slit-thickness skin graft healed within 2 weeks unlike the conventional dressing group where only about 18% of the patients had complete healing about the same time [12]. The ability to shorten patients´ hospital stay will go a long way in reducing the cost of care and also enhancing patients´ return to work. This is especially because studies have demonstrated that negative pressure dressing is now achieved with locally available materials which give very similar results as the expensive customized VAC^R^ which was rather far more expensive, about 50 times the cost of conventional dressing method [11,13]. The study also shows that patients in the negative pressure dressing group were more satisfied with the outcome of the grafting compared to the patients who had traditional wound dressing. All the 31 cases in the negative pressure groups gave the highest rating of satisfaction which was designated ‘very satisfied’ compared to the traditional dressing in which only 3 cases were ‘very satisfied’. This implies more than 10 times satisfaction rate with the negative pressure dressing. In the traditional dressing therefore the 9.68% had highest satisfaction rate, while 70.97% were moderately satisfied and 19.35% were just fairly satisfied. None was completely dissatisfied.

The p-value was less than 0.05 and is therefore statistically significant. The satisfaction correlates with relative less pain, the improved graft takes as well as the absence of complications and the ultimate quality of the graft. As earlier established the negative pressure could significantly reduce complications and improve the graft take. It would ultimately lead to better patients´ satisfaction. This agrees with the position of Hsiao *et al*. in which they showed that patients with negative pressure dressing were more satisfied than their counterparts with petroleum gauze dressing. They showed 78.9% of patients were very satisfied in the negative pressure dressing compared to their counterparts in the petroleum gauze dressing in which only 7.1% rated the procedure as very good. The 100% of negative pressure dressing group rated the procedure as comfortable, convenient and recommendable as opposed to the petroleum gauze group where only 16.7% rated it as both comfortable and recommendable, 0% rated it as convenient. They also observed less pain in the negative pressure group compared to the conventional dressing group [14]. Pyo *et al*. also observed better patient satisfaction which was as a result of the increased mobility after negative pressure dressing and the perceived increased effectiveness of the medical team due to the greater ease of dressing application compared to the conventional method [15]. It has also been noted that patients were more comfortable with less need for dressing change, lower cost of nursing care and less risk of cross infection [16]. All these would contribute to patients´ satisfaction with the negative pressure dressing.

## Conclusion

Prolonged hospital stay in chronic leg wounds is financially, socially and mentally demanding on patients. Efforts toward reducing this have a lot of positive impact on the patient. Therefore, negative pressure dressing which reduces length of stay both in the pre-grafting and post-grafting periods consequently increase patient satisfaction. This could be achieved with very cheap locally adapted negative pressure dressing apparatus with very commensurate outcome.

### What is known about this topic

- Negative pressure dressing shortens with bed preparation with customized VAC^R^;
- It improves graft take.

### What this study adds

- Locally adapted negative pressure dressing is cheaper, achieves same faster wound bed;
- In addition, it shortens graft healing time and overall hospital stay with cheap readily available set up;
- Comparison of the locally adapted negative pressure apparatus with conventional dressing is new.
